# Study on the effect of koumiss on the intestinal microbiota of mice infected with *Toxoplasma gondii*

**DOI:** 10.1038/s41598-022-05454-x

**Published:** 2022-01-24

**Authors:** Xinlei Yan, Wenying Han, Xindong Jin, Yufei Sun, Jialu Gao, Xiuli Yu, Jun Guo

**Affiliations:** Food Science and Engineering College of Inner Mongolia Agricultural University, Hohhot, 010018 China

**Keywords:** Infection, Drug development, Public health

## Abstract

*Toxoplasma gondii* is a worldwide food-borne parasite that can infect almost all warm-blooded animals, including humans. To date, there are no effective drugs to prevent or eradicate *T. gondii* infection. Recent studies have shown that probiotics could influence the relationship between the microbiota and parasites in the host. Koumiss has been used to treat many diseases based on its probiotic diversity. Therefore, we explored the effect of koumiss on *T. gondii* infection via its effect on the host intestinal microbiota. BALB/c mice were infected with *T. gondii* and treated with PBS, koumiss and mares’ milk. Brain cysts were counted, and long-term changes in the microbiota and the effect of koumiss on gut microbiota were investigated with high-throughput sequencing technology. The results suggested that koumiss treatment significantly decreased the cyst counts in the brain (*P* < 0.05). Moreover, *T. gondii* infection changed the microbiota composition, and koumiss treatment increased the relative abundance of *Lachnospiraceae* and *Akkermansia muciniphila*, which were associated with preventing *T. gondii* infection. Moreover, koumiss could inhibit or ameliorate *T. gondii* infection by increasing the abundance of certain bacteria that control unique metabolic pathways. The study not only established a close interaction among the host, intracellular pathogens and intestinal microbiota but also provided a novel focus for drug development to prevent and eradicate *T. gondii* infection.

## Introduction

*Toxoplasma gondii* is a worldwide intracellular protozoan parasite that can infect almost all cell types, including humans, livestock, companion animals and wildlife^1^. It is estimated that nearly one-third of the human population has been exposed to *T. gondii*^2^. Infection may occur mainly by ingesting raw vegetables and water containing sporulated oocysts or uncooked meat containing tissular cysts or by vertical transmission^3,4^. Toxoplasmosis can cause health problems in humans, such as encephalitis, pneumonitis and myocarditis, and even result in death in immunodeficient patients^2^. Moreover, infection can cause foetus miscarriage, congenital malformations and other symptoms during pregnancy^5^. Once a human is infected with *T. gondii*, tachyzoites massively proliferate and subsequently persist for a long time as dormant cysts, preferentially in the brain and muscles^6^.

Chronic infection was previously considered benign^7^, but recent studies have shown that it is responsible for neurological disorder diseases, such as bipolar disease, schizophrenia and epilepsy, although the causal relationships are unclear^8–10^. In addition, dormant cysts can be reactivated and lead to severe consequences, including encephalitis, when host immunity is defective^11^. Nevertheless, effective drugs that prevent or eradicate *T. gondii* tissue cyst formation have not been identified, although 50 compounds affecting over 20 different pathways or mechanisms were tested over the last three decades^7^. Therefore, the identification of a significant method for treating chronic *T. gondii* infection holds great promise.

Recently, the gut microbiota, which can affect the gastrointestinal system and other distal organs in the body, including the brain, has attracted more attention. Many studies have made great progress by establishing correlations between intestinal microbiota and diseases, such as hepatic encephalopathy, Alzheimer’s disease and schizophrenia, and treatment effects, such as antidepressant effects^12–15^. Many studies have shown that there is a close interaction among parasites, microbial communities, and host immune responses^16^. Furthermore, probiotics can disrupt the relationship between the parasite and associated microbiota by increasing the gut microbiota diversity to change the microbiota composition^17^. However, to date, no study has linked gut microbiota with toxoplasmosis treatment.

Koumiss is a traditional fermented drink product of nomadic people in central Asia, which is made by fermenting mares’ milk with lactic acid bacteria and yeasts^18^. The rich microbial microbiota caused by spontaneous fermentation determines its nutritional and medical value. Guo et al. separated 6 bacterial phyla, represented by 126 genera and 49 species, and 3 fungal phyla, represented by 59 genera and 57 species, from 11 artisanal koumiss samples^19^. In China, koumiss was the earliest beverage used in food therapy with an obvious auxiliary treatment effect in digestive diseases^20^. In addition, koumiss has been widely used in treating hyperlipidemia and chronic atrophic gastritis and for promoting immunomodulation and anti-inflammatory effects because of its probiotic diversity^21–23^.

In this study, we explored the possibility that koumiss consumption treats or alleviates toxoplasmosis by modulating the intestinal microbiota of mice chronically infected by *T. gondii*. After mice infected by *T. gondii* were treated with Koumiss, the brain cysts were counted, and the 16S rRNA gene sequencing of the gut microbiota was determined with high-throughput sequencing technology. This study reveals the effects of koumiss consumption in treating toxoplasmosis by establishing the relationship between *T. gondii* and the gut microbiota in the host and provides a new idea for drug development.

## Results

### Infection of mice with *T. gondii* and cyst counting

**Figure 1:**
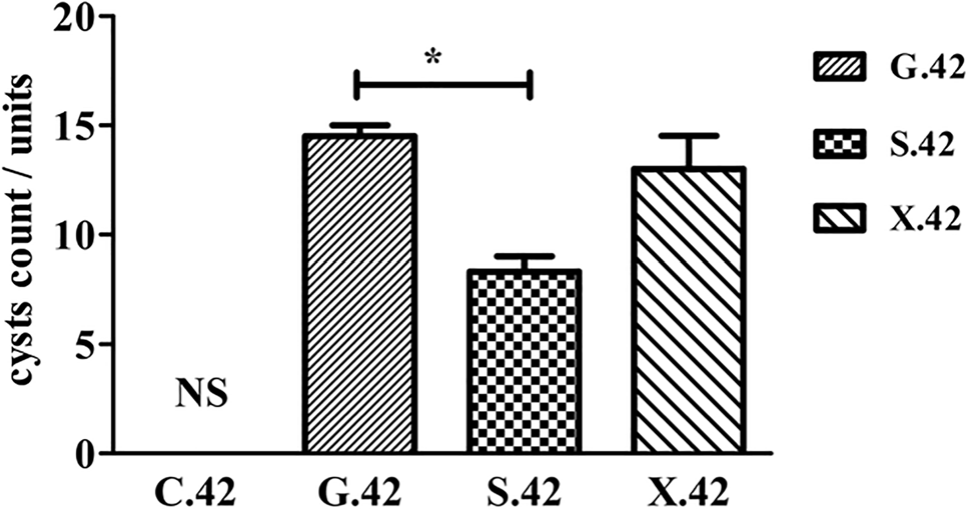
The cyst counts of the C, G, S and X groups at 42 dpi.

The feces of the C, G, S and X groups were collected on 7 dpi, 14 dpi, 21 dpi, 28 dpi, 35 dpi, and 42 dpi, respectively. Moreover, the brain tissue of the C, G, S and X groups at 42 dpi was collected and homogenized to count cysts. As shown in Fig. 1, the cyst counts in the brains of the G, S and X groups were 14.5/units, 8.33/units, and 13/units, respectively. The cyst counts of the S group were significantly lower than those of the G group (*P* < 0.05), indicating that koumiss may have a regulating effect on *T. gondii* infection.

### Different treatment methods changed the abundance of the intestinal microbiota in mice

**Figure 2:**
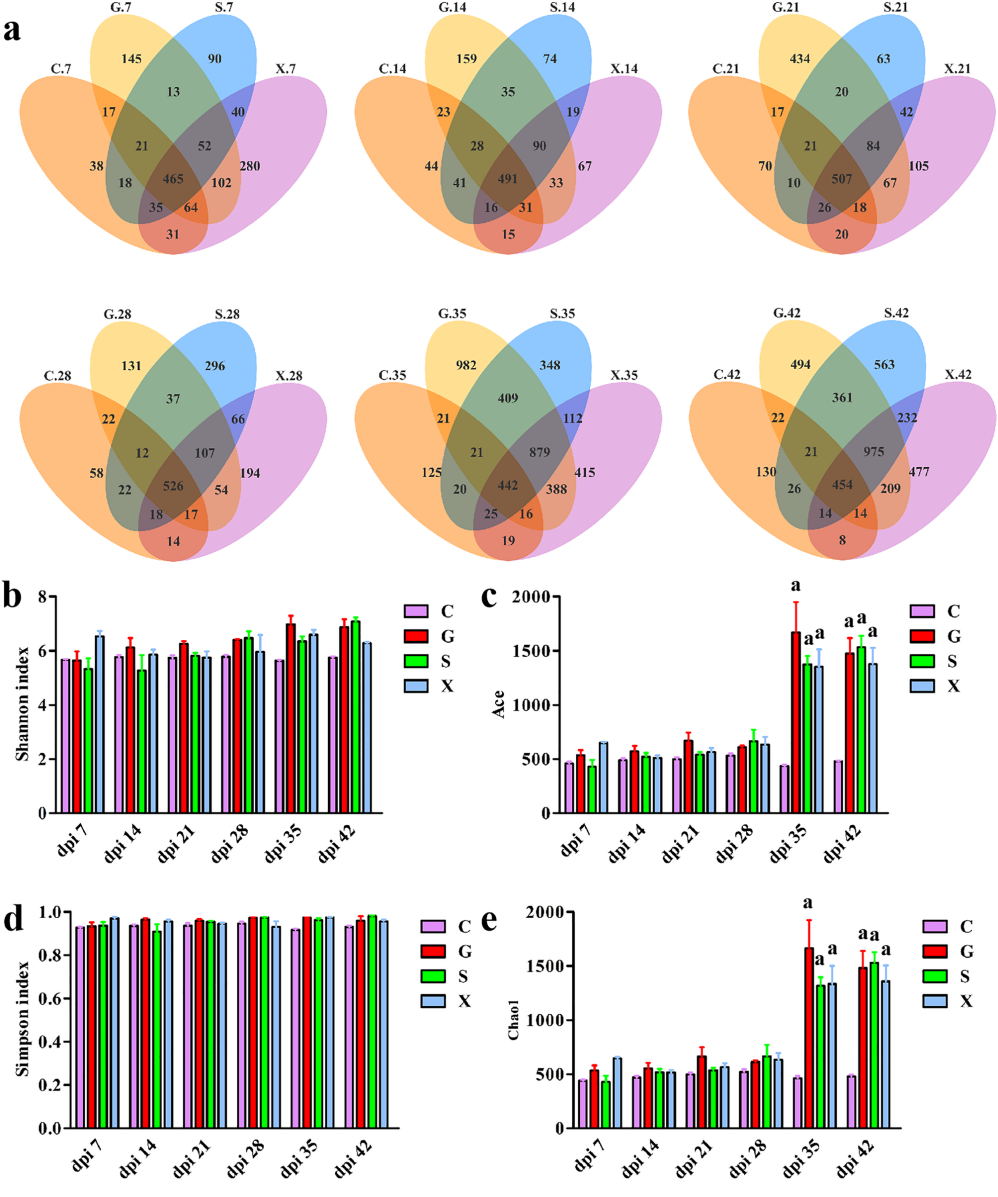
Different treatment methods change the intestinal microbiota diversity of mice. (**a**) Venn diagram showing OTUs of the C, G, S and X groups at 7 dpi, 14 dpi, 21 dpi, 28 dpi, 35 dpi and 42 dpi. Overlapping parts represent shared OTUs among groups. (**b**) Shannon, (**c**) Ace, (**d**) Simpson, and (**e**) Chao1 indices in α-diversity analyses. **a** indicates *P* < 0.001.

In the present study, we determined the 16S rRNA gene sequence of the gut microbiota in the C, G, S and X groups at 7 dpi, 14 dpi, 21 dpi, 28 dpi, 35 dpi and 42 dpi. All effective tags of samples were clustered with 97% identity into OTUs, which were used to better analyze species composition. The OTUs of overlapping parts peaked at 28 dpi, indicating the most similarity in different groups. However, the total number of OTUs at 35 dpi was significantly higher than before, and greater differences in species composition were observed among the groups (Fig. 2a). As shown in Fig. 2b, d, the Shannon and Simpson indices, which describe species diversity and uniformity, were not significantly different among the groups. In addition, the Ace and Chao1 indices of the G, S and X groups at 35 dpi and 42 dpi were dramatically higher than those of the C group (*P* < 0.001) (Fig. 2c, e). The results showed that the G, S and X groups had higher microbiota richness at 35 dpi and 42 dpi, which was consistent with the Venn diagram results.

### The community structure of microbiota at phylum level

**Figure 3:**
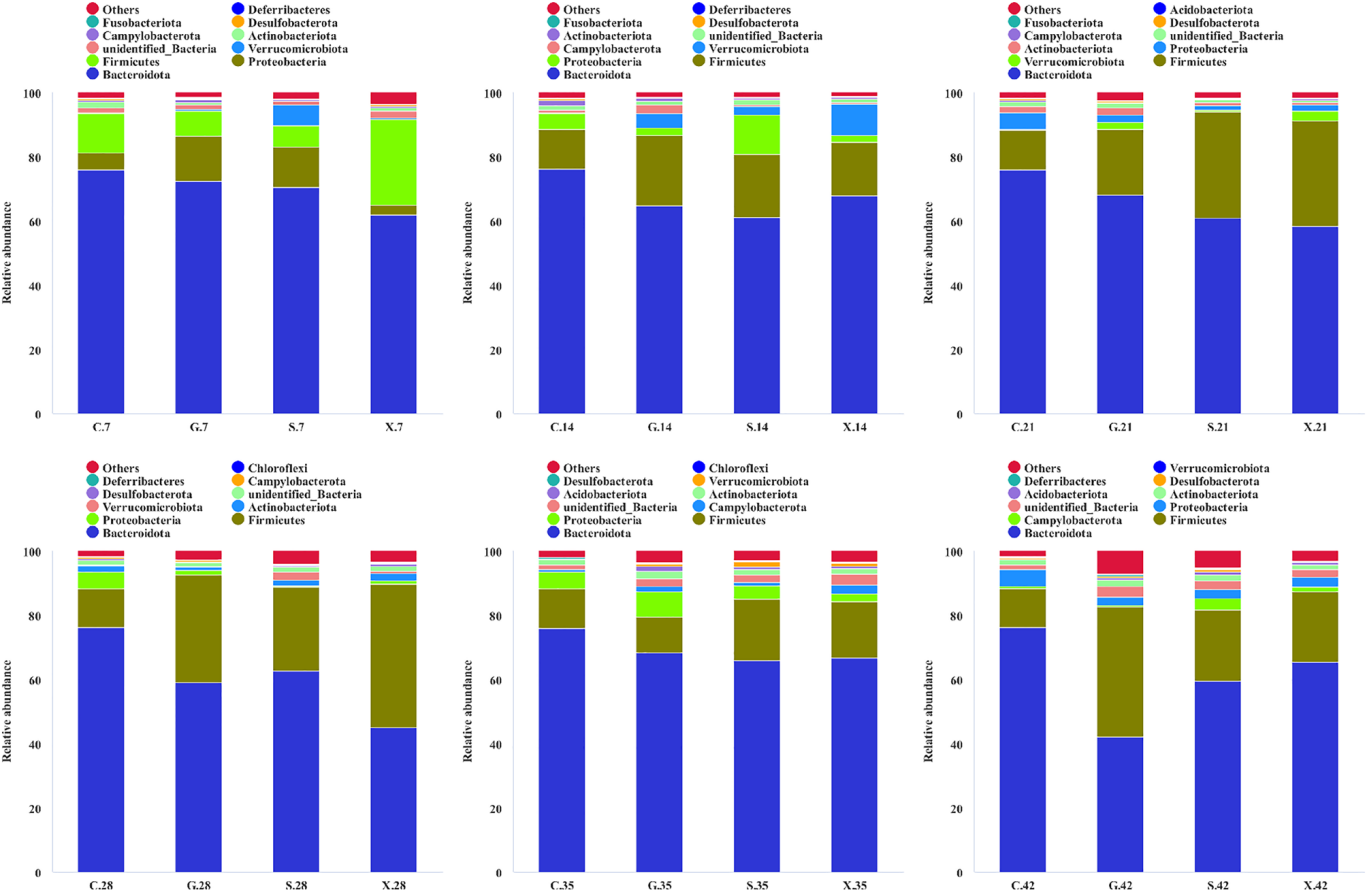
Community structure of microbiota at the phylum level.

The community structure of samples at different taxonomic levels on the basis of OTUs, including phylum, class, order, family, genus and species, was determined. In this study, 59 phyla, represented by 745 genera and 357 species, were identified. The composition of the microbiota at the phylum level, whose abundances were ranked in the top 10, was mainly analyzed. The community structure of the C group on 7 dpi included *Bacteroidota* (76.1%), *Firmicutes* (12.4%), *Proteobacteria* (5.2%), *Actinobacteriota* (1.9%) and others (Fig. 3). After infection with *T. gondii*, *Bacteroidota* was less abundant in all treatment groups than in the C group, although it had the highest abundance. Nevertheless, the abundance of *Firmicutes* exhibited an obvious increase in the G, S and X groups until 28 dpi. The G group exhibited the maximum increase from 7.8 (7 dpi) to 40.5% (42 dpi). On 28 dpi, the abundance of *Fusobacteriota* decreased to nearly 0 in the G, S and X groups. Moreover, the abundance of *Actinobacteriota* maintained a stable increase in the S group and was close to that in the C group at 42 dpi.

### *Toxoplasma gondii* infection influenced the gut microbiota composition of the mice

**Figure 4:**
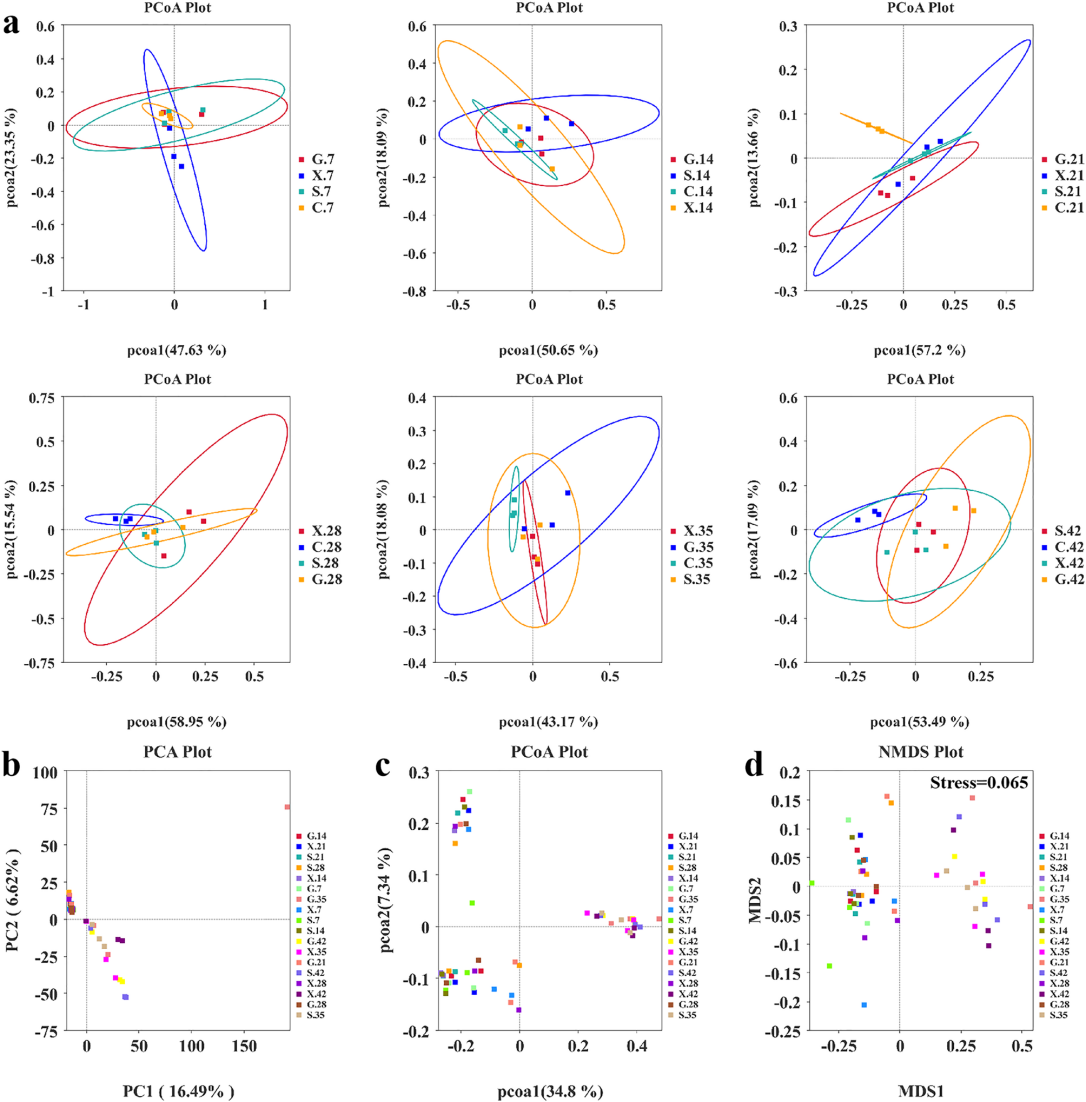
Alterations in gut microbiota caused by *T. gondii* infection. (**a**) PCoA of the weighted UniFrac values for the C, G, S and X group on 7 dpi, 14 dpi, 21 dpi, 28 dpi, 35 dpi and 42 dpi. (**b**) PCA and (**c**) PCoA of the unweighted UniFrac values for the G, S and X groups at 7 dpi, 14 dpi, 21 dpi, 28 dpi, 35 dpi and 42 dpi. (**d**) NMDS plot showing the differences in gut microbiota composition based on the unweighted UniFrac distance. A stress < 0.2 means that the NMDS plot can accurately reflect differences among samples.

Multivariate statistical methods based on UniFrac distance were used to analyze the changes in the gut microbiota in the mice infected by *T. gondii*. The gut microbiota composition of the G, S and X groups was markedly distinct from that of the C group, which was clustered separately beginning on 21 dpi (Fig. 4a). The results suggested that *T. gondii* infection resulted in alterations in gut microbiota composition. Moreover, the PCA plot demonstrated that the microbiota community structure of the G, S and X groups at 35 dpi and 42 dpi was separated from the G, S and X groups at 7, 14, 21 and 28 dpi by PC1 (16.49% of the explained variance) (Fig. 4b). There was also a separation of 35 and 42 dpi from 7, 14, 21 and 28 dpi in the G, S and X groups by PCoA1 (34.8% of the explained variance) (Fig. 4c). NMDS was applied to overcome the shortcomings of PCA and PCoA and determine the species information of samples. The same result was observed on the two-dimensional plane in the form of points, indicating more significant variation in the intestinal microbiota composition caused by *T. gondii* infection over time (Fig. 4d).

### The impact of Koumiss treatment on the intestinal microbiota of mice infected with *T. gondii*

**Figure 5:**
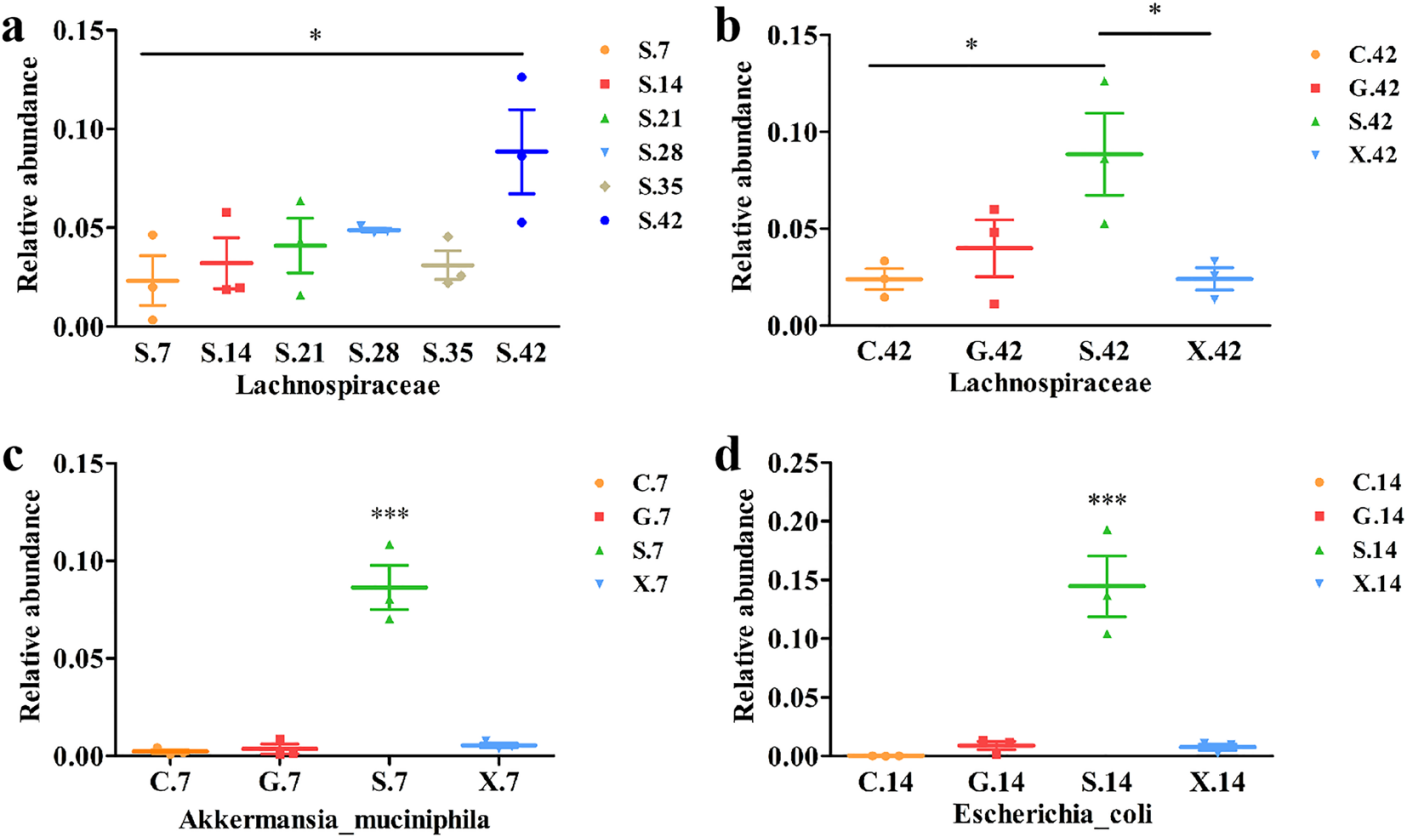
The impact of Koumiss treatment on the intestinal microbiota of mice infected with *T. gondii*. (**a**) The relative changes in *Lachnospiraceae* abundance in the S group on 7 dpi, 14 dpi, 21 dpi, 28 dpi, 35 dpi and 42 dpi. (**b**) The relative abundance of *Lachnospiraceae* in the C, G, S and X groups at 42 dpi. (**c**) The relative abundance of *Akkermansia muciniphila* in the C, G, S and X groups at 7 dpi. (**d**) The relative abundance of *Escherichia coli* in the C, G, S and X groups at 14 dpi. *P < 0.05; ***P < 0.001.

To study the impact of koumiss on the microbiota of mice infected with *T. gondii*, we compared the differences in the species among different groups using Tukey’s test. Koumiss treatment changed the intestinal microbiota composition in mice infected with *T. gondii*, such as the abundances of *Actinobacteriota*, *Lactobacillaceae* and *Acetobacter*. The most striking finding was that the relative abundance of *Lachnospiraceae* continuously increased after the mice were orally gavaged with koumiss (Fig. 5a). There was a significant difference between 7 and 42 dpi (*P* < 0.05). Furthermore, there were significant differences on 42 dpi between the S and C group (*P* < 0.05), S and X group (*P* < 0.05), respectively (Fig. 5b). More importantly, the relative abundance of *Akkermansia muciniphila* in the S group on 7 dpi was dramatically different from that in the C, G and X groups (*P* < 0.001) (Fig. 5c) Moreover, there was a significant increase in the relative abundance of *Escherichia coli* in the S group compared with that in the C, G and X groups on 14 dpi (*P* < 0.001) (Fig. 5d).

### Koumiss treatment caused changes in the microbiota functional profile

**Figure 6:**
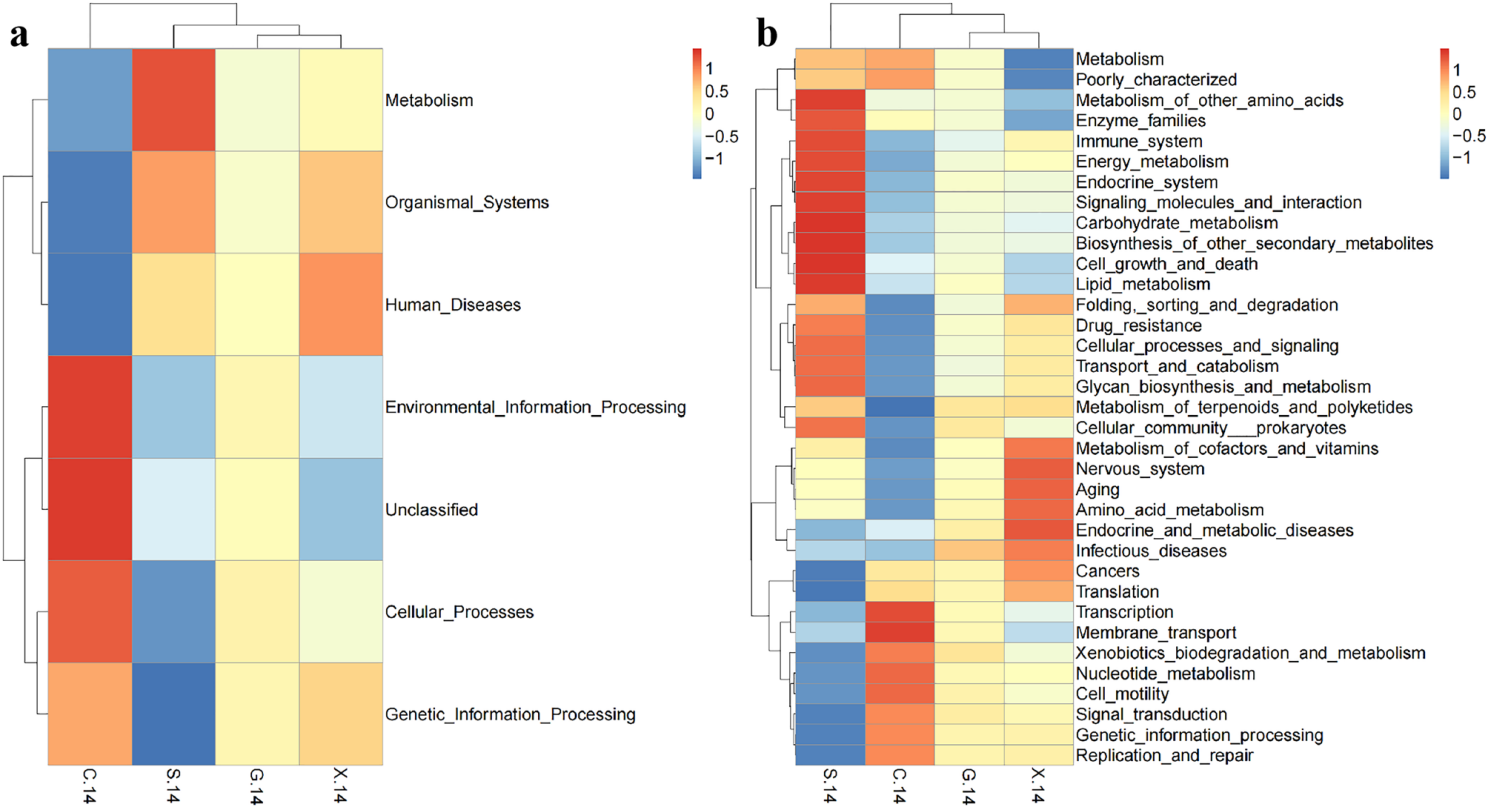
Heatmaps of KEGG functional classification of different groups at 14 dpi in Level 1 (**a**) and Level 2 (**b**).

Tax4Fun analysis was carried out to understand the differences in the function of the intestinal microbiota among the treatment groups. The Tax4Fun software package is well-known for its high accuracy in predicting the function of environmental samples, such as the intestine and soil. The key is to compare 16S rRNA gene sequencing data with the KEGG database to achieve functional annotation. The results showed that at 14 dpi, the mice in the S group had more bacteria than those in the C, G and X groups that functioned in metabolism, including glycan biosynthesis and metabolism, carbohydrate metabolism, energy metabolism, biosynthesis of other secondary metabolites and endocrine metabolism (Fig. 6a, b). There was also an obvious increase in cellular processes and signaling, drug resistance, immune system and signaling molecules and interactions in the S group at 14 dpi. However, membrane transport in the S group at 14 dpi showed a downward trend compared with that in the C group (Fig. 6b).

## Discussion

*Toxoplasma gondii* is a food-borne parasite found worldwide that can infect almost all warm-blooded animals, including humans^24^. Chronic *T. gondii* infection often results in few clinical symptoms in immunocompetent individuals. However, some studies have suggested that it can result in changes in immunity, brain transcriptomic signatures, microbiota, and even behavior and personality in the host^6,25,26^. Some studies have revealed that a healthy intestinal microbiota is a complex ecological community that can carry out critical physiological functions with the intestinal mucosa of the host^27^. Moreover, changes in the gut microbiota composition can prevent or alleviate intestinal protozoan infection and affect the ultimate outcomes of parasitic diseases. There is also a close connection among the host, microbiota and pathogens. An interesting example is that a certain gut microbiota composition in the host can impede gut colonization by helminths or reduce the colonization time^28^. To date, there is no study that associates the host microbiota with the treatment of *T. gondii* infection. Shao et al. only discussed the changes in the intestinal microbiota 13 days and 21 days after infection with *T. gondii*^25^. In this study, we investigated not only the effect of koumiss treatment on the intestinal microbiota of mice chronically infected with *T. gondii* but also the long-term changes in the gut microbiota caused by *T. gondii* infection, including the changes that occur in the acute infection stage, chronic infection stage and cyst formation stage.

In this study, we determined the 16S rRNA gene sequences of the gut microbiota of the C, G, S and X groups at 7 dpi, 14 dpi, 21 dpi, 28 dpi, 35 dpi and 42 dpi. The intestinal microbiota community structure at the phylum level was mainly analyzed. *Bacteroidota* was the most abundant phylum in the C, G, S and X groups, and the relative abundance of *Bacteroidota* decreased after infection with *T. gondii*. There was an obvious increase in the relative abundance of *Firmicutes* in all the experimental groups, which was different from a previous study^25^. In a previous study, mice were infected with 100 purified sporulated oocysts of the ME49 strain, which may have caused inflammation in the intestine and was associated with a low F/B (*Firmicutes*/*Bacteroidota*) index^29^. In this study, mice were inoculated with 3 cysts to establish chronic infection, and no intestinal inflammation was observed (data not shown), but this treatment resulted in a high F/B index. The gut-brain axis plays an important and complex bidirectional role in communication between the intestine and the central nervous system (CNS) and is involved in different pathways and systems, including the autonomic and enteric nervous system, the endocrine system, the hypothalamic–pituitary–adrenal axis (HPA), the immune system, and the regulation of the microbiota and its metabolites^30–32^. Therefore, we considered that one of the reasons that explains our results is that parasites might influence the intestinal microbiota through the gut-brain axis. Moreover, this parasite is distributed all around the host during chronic infection, which makes the mechanism more difficult to understand; this is an interesting phenomenon worthy of further study.

In addition, we found that *T. gondii* infection dramatically changed the community structure of the microbiota. The C group and other treatment groups showed an obvious separation in the PCoA plot at 21 dpi, indicating that *T. gondii* infection would alter the intestinal microbiota composition, which was consistent with previous studies^25^. Nevertheless, the C group was not separately clustered from the other treatment groups at 14 dpi. This result may have occurred because a large number of parasites had appeared in the brain. The strains of *T. gondii* and the inoculation dose and time may affect the distribution of *T. gondii.* Zenner et al. confirmed that *T. gondii* first reached the mesenteric lymph nodes (MLNs) through the intestinal wall, then reached spleen, and finally reached other organs^33^. Saeij et al. thought that a virulent strain of *T. gondii* first appeared in the head and neck, whereas an attenuated strain appeared preferentially in the abdomen^34^. The changes in the microbiota composition caused by *T. gondii* in the brain were also associated with the gut-brain axis. Interestingly, there was also an apparent separation of 35 and 42 dpi from 7, 14, 21 and 28 dpi in the G, S and X groups, which indicated that the community structure of the gut microbiota could change over time during *T. gondii* infection. The results are shown in the PCA plot, PCoA plot, NMDS plot and Venn diagrams. The Ace and Chao1 indices of the G, S and X groups were obviously increased at 35 and 42 dpi. The results demonstrated that there was an obvious increase in the gut microbiota diversity during *T. gondii* cyst formation and that this increased diversity persisted for a long time. Traditionally, pathogen infection is thought to decrease the microbiota richness. However, the results of this study were completely opposite of that previous theory. This discovery has important implications for understanding the connection between pathogens and the intestinal microbiota in the host.

The effect of koumiss treatment on intestinal microbiota was also discussed in this study. It should be noted that the infected mice treated with koumiss were not significantly different from the untreated mice in terms of OTUs, the Shannon index or the Simpson index. However, we still found that koumiss treatment altered the relative abundance of certain bacteria, such as *Actinobacteriota*, *Lactobacillaceae* and *Acetobacter*. The most important alteration was that the relative abundance of *Lachnospiraceae* continuously increased after the mice infected with *T. gondii* were orally gavaged with koumiss. There were significant differences in the relative abundance of *Lachnospiraceae* between the S and C group, the S and X group at 42 dpi. *Lachnospiraceae* are a family of obligate anaerobic bacteria that are abundant in healthy humans. *Lachnospiraceae* can facilitate colonization resistance against intestinal pathogens by producing the short-chain fatty acids (SCFAs) acetate and butyrate^35^, converting primary to secondary bile acids^36^ or producing lantibiotics^37^. Therefore, it was thought that the reduction in the numbers of cysts in the brain in the S group, a key metric indicating decreased parasitic burden during chronic *T. gondii* infection, may be associated with a high level of *Lachnospiraceae*. Moreover, the results published in *Science* showed that *Lachnospiraceae* can allow mice to survive after receiving a lethal dose of total body radiation by promoting hematopoiesis restoration and gastrointestinal repair^38^. More importantly, *Lachnospiraceae* abundances can be altered by changes in diet^39^. This will be further explored for the treatment of *T. gondii* infection. Furthermore, we expectedly found that the relative abundance of *A. muciniphila* in the S group at 7 dpi dramatically differed from that in the C, G and X groups (*P* < 0.001). *A. muciniphila* is an emerging beneficial bacterium that controls the essential regulatory system of glucose and energy metabolism^40^. In addition, *A. muciniphila* can also decrease blood lipid levels and affect metabolic modulation, immune response regulation, and gut health maintenance^41–44^. Interestingly, it was also found that *A. muciniphila* could facilitate the expulsion of intestinal *Trichinella spiralis* through the development of goblet cell hyperplasia and secretion of mucus^45,46^. Although *A. muciniphila* was isolated from human feces in 2004, the relationship between *A. muciniphila* and intracellular pathogens is still unknown. This study provides a novel focus for establishing connections between these species through the intestinal microbiota.

Koumiss treatment also influenced the function of the microbiota in the intestine. The results showed that at 14 dpi, the mice in the S group had more bacteria than those in the C, G and X groups that functioned in metabolism, such as carbohydrate metabolism, lipid metabolism and amino acid metabolism. Host metabolites can not only promote the growth of *T. gondii* but also directly govern its development and differentiation^47^. The glycolysis pathway is very important for the *T. gondii* tachyzoite stage, and this pathway not only provides a carbon source for fatty acid synthesis but also drives *T. gondii* invasion of host cells^48^. After hosts are infected by *T. gondii*, dendritic cells and macrophages drive an immune response but also improve the dependence of metabolism on glycolysis, which supports *T. gondii* growth^49^. *A. muciniphila* may inhibit *T. gondii* growth by regulating glucose and energy metabolism. Furthermore, research has suggested that *T. gondii* infection increases the relative abundance of *Escherichia coli* in the intestine^50^. This may be the reason why the relative abundance of *Escherichia coli* was higher in the S group than in the C, G and X groups at 14 dpi. Lipids also participate in innate immunity and may play an important role in controlling *T. gondii* infection^51^. *Lachnospiraceae* may facilitate colonization resistance against intestinal pathogens through the lipid metabolism pathway. *T. gondii* infection also interferes with arginine uptake, synthesis and metabolization in the host cell. A study demonstrated that *T. gondii* induced the integrated stress response (ISR) by depleting arginine from host cells, which caused increased expression of the host cationic amino acid transporter CAT1 to ensure a constant supply of arginine^52^. Compared with that in uninfected cells, the expression of arginine succinate synthase 1 (ASS) in *T. gondii-*infected cells was upregulated by fivefold^53^. A study also indicated that *T. gondii* differentiated toward bradyzoites when essential nutrient supplies, such as arginine and cysteine, were limited^54^. Moreover, we strikingly found that the S group had fewer bacteria that function in membrane transport at 14 dpi. *T. gondii* secretes rhoptries (ROPs) and dense granules (GRAs) that interfere with biological processes in host cells, resulting in slight evasion of host cell autonomous defense mechanisms^55^. Secreted proteins are also regarded as nonspecific small molecule channels that facilitate the flux of metabolites between the parasitophorous vacuole (PV) space and the host cytoplasm^56^. Therefore, koumiss may inhibit *T. gondii* energy supplementation by reducing metabolite transport. In addition, it was found that at 14 dpi, the S group had more bacteria in the organismal system, including the immune system, which were beneficial for preventing *T. gondii* infection.

In conclusion, we investigated brain cyst counts and explored long-term changes in the microbiota and the effect of koumiss treatment on the microbiota in mice infected with *T. gondii*. Strikingly, we found that koumiss treatment significantly reduced brain cyst counts in mice infected with *T. gondii*. Moreover, *T. gondii* infection altered the intestinal microbiota community structure, whereas koumiss treatment could enhance the relative abundance of *Lachnospiraceae* and *A. muciniphila*; this effect of ameliorating *T. gondii* infection occurred via special metabolic pathways. The study established a close interaction among the host, intracellular pathogens and intestinal microbiota and provided a novel direction for preventing and eradicating *T. gondii* infection.

## Materials and methods

### Animals and parasites

One hundred and forty-four 6-week-old female BALB/c mice, weighing 15–18 g, were purchased from SPF (Beijing) Biotechnology Co., Ltd. The mice (three per cage) were given ad libitum access to food and water and housed under a 12 h light/dark cycle. The animal protocols were reviewed and approved by the Inner Mongolia Agricultural University Laboratory Animal Welfare and Animal Experimental Ethical Inspection Committee (approval No.: 2020-037). All experiments were performed in accordance with relevant guidelines and regulations. And the study was conducted in compliance with the ARRIVE (Animal Research: Reporting of In Vivo Experiments) guidelines^57^. The *T. gondii* Prugniaud strain was obtained from the National Animal Protozoa Laboratory of China Agricultural University. After 1 week of acclimation, the mice were randomly divided into Control group (C, n = 36), Infection group (G, n = 36), Koumiss group (S, n = 36) and Mare milk group (X, n = 36). Then, the G, S and X groups were infected orally with 200 µL sterile phosphate buffered solution (PBS) containing 3 tissular cysts, while the C group was infected orally with the same volume of sterile PBS alone. The G, S and X groups were orally gavaged with 100 µL PBS, koumiss and mares’ milk at two-day intervals. Koumiss samples and fresh mare milk samples obtained from the Abaga banner of Xilin Gol League were used in their original form.

### Samples collection and cyst counting

Six mice were selected randomly from each group and individually placed in clean and sterile cages. Then, the feces were collected aseptically within two hours, and the mice were euthanized by cervical dislocation. Mice of the C, G, S and X group were euthanized at 7 days post infection (dpi), 14 dpi, 21 dpi, 28 dpi, 35 dpi, and 42 dpi. The feces were treated with liquid nitrogen and stored at − 80 °C. The mice were sacrificed at 42 dpi, the brains were homogenized, and the cysts were counted three times as described previously^58^.

### DNA extraction and PCR amplification

Genomic DNA was extracted from feces using the cetyltrimethylammonium ammonium bromide (CATB) method. The purity and concentration of the nucleic acids were determined by 1% agarose gel electrophoresis. The 16S rRNA genes (V3–V4 region) were amplified with specific primers with barcodes. Amplification was performed with primers with the following sequences: forward primer 515F (5′-GTGCCAGCMGCCGCGGTAA-3′) and reverse primer 806R (5′-GGACTACHVGGGTWTCTAAT-3′). Thermal cycling consisted of initial denaturation at 98 °C for 1 min, 30 cycles of denaturation at 98 °C for 10 s, annealing at 50 °C for 30 s, and elongation at 72 °C for 30 s, and a final elongation step at 72 °C for 5 min. The PCR products were detected by 2% agarose gel electrophoresis and purified with a Qiagen Gel Extraction Kit (Qiagen, Germany).

### Library construction and sequencing

Following library construction by the TruSeq^®^ DNA PCR-Free Sample Preparation Kit (Illumina, USA), the library was sequenced on the Illumina NovaSeq sequencing platform, and 250 bp paired-end reads were generated. The measurement was repeated three times for each sample. The reads were merged with FLASH (VI.2.7, http://ccb.jhu.edu/software/FLASH/) and filtered rigorously according to the QIIME (V1.9.1, http://qiime.org/scripts/split libraries fastq.html/) quality control process^59^. After the tags were compared with the reference database (Silva database, https://www.arb-silva.de/), chimera sequences were removed, and the effective tags were finally obtained.

### Biological information analyses

All effective tags of samples were clustered with 97% identity into operational taxonomic units (OTUs) by the Uparse algorithm (V7.0.1001, http://www.drive5.com/uparse/). The taxonomic information was obtained by species annotation analysis, and the community composition of samples was counted individually at each classification level, including the Kingdom, Phylum, Class, Order, Family, Genus and Species levels. The least amount of data in the sample was used as the standard for homogenization, which was the basis for subsequent alpha diversity analysis and beta diversity analysis. Principal component analysis (PCA), a method of variance decomposition based on Euclidean distances, was used to reduce the dimension of multidimensional data and extract the most important elements in the data^60^. In addition, to overcome the shortcomings of linear models, such as PCA and principal coordinates analysis (PCoA), nonmetric multidimensional scaling (NMDS) was also applied to determine the species information of the samples, and the results are expressed in the form of points on the two-dimensional plane^61^.

### Statistical analysis

Alpha diversity index analyses of the samples were performed with QIIME by calculating the Chao1, Shannon, Simpson and Ace indices. Shannon and Simpson indexes were used to describe the species diversity and equitability. The higher the community diversity was, the greater the Shannon and Simpson indexes were. Chao1 and Ace indexes reflected on the number of species and OTUs respectively. Among all the indices at the level of Alpha-diversity, Shannon index presents remarkable characteristics. It was worth noting that Shannon and Simpson indexes would decrease when the relative abundance of species in a community exceeded a certain limitation. The limit was about 0.67 for the Simpson index and about 0.7 for the Shannon index. QIIME also served to calculate UniFrac distance. R software was used to draw PCA, PCoA and NMDS diagrams. GraphPad Prism software was also used for data analyses and plots. Kyoto Encyclopedia for Genes and Genomics (KEGG) classifications were performed with the Tax4Fun software package. Statistically significant differences in species composition between groups were determined using Tukey’s test. *P* < 0.05 indicated a significant difference.
